# Efficacy of Teprenone for Prevention of NSAID-Induced Gastrointestinal Injury: A Systematic Review and Meta-Analysis

**DOI:** 10.3389/fmed.2021.647494

**Published:** 2021-04-07

**Authors:** Chongxiang Zhao, Jingwu Wang, Qiang Xiao

**Affiliations:** Department of Gastroenterology, Zaozhuang Hospital of Traditional Chinese Medicine, Zaozhuang, China

**Keywords:** teprenone, aspirin, NSAID, complication, ulcer, gastrointestinal

## Abstract

**Background:** The study aimed to conduct a systematic review and meta-analysis comparing the efficacy of teprenone with control or other drugs for reducing the incidence of gastrointestinal (GI) adverse events in patients receiving long-term non-steroidal anti-inflammatory drugs (NSAIDs).

**Methods:** Databases of PubMed, Embase, BioMed Central, CENTRAL, and Google Scholar were searched up to November 10th, 2020 for randomized controlled trials (RCTs) comparing teprenone with control or other drugs. A random-effects model was used for the meta-analysis. Grading of Recommendations Assessment, Development, and Evaluation (GRADE) tool was used for assessing the certainty of evidence.

**Results:** Seven RCTs were included. Six compared teprenone with control and one with famotidine. Meta-analysis indicated a statistically significant reduced risk of GI ulcers in patients receiving teprenone as compared to control after 12 weeks/3months (RR 0.37 95% CI 0.17, 0.18 *I*^2^ = 0% *p* = 0.01). Pooled data of three open-label studies indicated statistically significant reduction of GI symptoms in patients on teprenone as compared to control at 6 months and 12 months, but not at 3 months. Comparing teprenone with control, our analysis indicated non-significant but a tendency of better reduction in Modified Lanza Score (MLS) with teprenone. The RCT comparing teprenone to famotidine demonstrated better reduction of MLS with famotidine. The certainty of evidence-based on GRADE was deemed to be low.

**Conclusion:** Low-quality evidence indicates a beneficial role of teprenone in preventing GI injuries in patients receiving long-term NSAIDs. Further high-quality RCTs comparing teprenone with placebo as well as other gastroprotective drugs are needed to strengthen current evidence.

## Introduction

Non-steroidal anti-inflammatory drugs (NSAIDs) are commonly prescribed in patients with cardiovascular, cerebrovascular, and rheumatoid disease (1, 2). Long-term treatment is usually required in such patients either for therapeutic purposes or for the prevention of adverse cardiovascular events like myocardial infarction and stroke. Among these drugs, low-dose aspirin has been the most commonly prescribed medication which has been in clinical use for several decades (1). It is known that even low doses of NSAIDs can result in gastrointestinal (GI) adverse events ranging from dyspeptic symptoms to ulcers and severe bleeding (3). However, the withdrawal of long-term NSAIDs can lead to increased mortality and other cardiovascular adverse events (4). Thus, a fine balance is to be maintained in clinical practice so that the benefits of long-term NSAIDs do not outweigh the associated risks.

The primary mechanism by which NSAIDs cause adverse GI events is by depletion of prostaglandin secretion and by direct topical injury (5). Since gastric acid is closely related to the pathogenesis of such adverse events, concurrent administration of acid-suppressing drugs like proton pump inhibitors (PPIs) and H2-receptor antagonists (H2RAs) is the commonly recommended strategy to prevent NSAID-induced GI side-effects (6). The efficacy of PPIs in reducing the incidence of GI adverse events is well-established in the literature (7). However, there have been concerns of increased risk of fractures, infections, and possible risk of dementia and renal disease with long-term use of PPIs (8). Furthermore, PPIs are expensive and may also affect platelet response thus reducing the efficacy of low-dose aspirin (9, 10). A recent narrative review on the use of PPIs for long-term treatment of gastroesophageal reflux has reiterated the efficacy of PPIs but has also highlighted the high-cost associated with long term treatment (10). Thus, an effective and safe alternative drug would enhance the spectrum of medications available to physicians for long-term prophylaxis against gastric injury.

Teprenone or geranylgeranylacetone is an anti-ulcerative drug that has been used for treating gastritis and gastric ulcers in several Asian countries. The drug is an acyclic isoprenoid compound that acts by activating heat shock protein 70 (HSP70). HSP70 is a cellular protective protein that prevents mucosal injury caused by agents like NSAIDs without affecting gastric acid secretion (11). Animal studies have demonstrated that teprenone can prevent NSAID-induced gastric as well as small intestinal injuries (12, 13). The efficacy of the drug has also been tested in several human trials, however, to the best of our knowledge there has been no effort to analyze and pool available evidence to guide clinical practice. Thus, the current study aimed to conduct a systematic literature search for studies comparing the efficacy of teprenone with control or other gastroprotective drugs for reducing the incidence of GI adverse events in patients receiving long-term NSAIDs and pool data for a meta-analysis.

## Materials and Methods

### Search Strategy

The review was conducted following the PRISMA statement (Preferred Reporting Items for Systematic Reviews and Meta-analyses) (14) and the Cochrane Handbook for Systematic Reviews of Intervention (15). Articles on the subject of the review were searched in the electronic databases of PubMed, Embase, BioMed Central, CENTRAL, and Google Scholar up to November 10th, 2020. Databases were searched from inception and without any language restriction. We used the following keywords in different combinations for the literature search: “teprenone,” “geranylgeranylacetone,” “gastrointestinal,” “gastric,” “small intestine,” “injury,” and “ulcer.” Supplementary Table 1 depicts the search strategy of the review. Two reviewers carried out the electronic search independent of each other. The primary search results were assessed initially by their titles and abstracts to identify citations requiring full-text analysis. The full-texts of the articles were reviewed by the two reviewers independently based on the inclusion and exclusion criteria. Any disagreements were resolved by discussion. Furthermore, we also hand-searched the bibliography of included studies for any missed references.

### Inclusion Criteria

To maintain clarity on the inclusion criteria, the PICOS (Population, Intervention, Comparison, Outcome, and Study design) guide was used to include studies. The following criteria were used for each domain:

*Population*: Adult patients prescribed long-term (at least 12 weeks or 3 months) NSAID therapy for any disease.
*Intervention*: Teprenone for the duration of NSAID therapy.
*Comparison*: Placebo or any other comparative drug (PPI or H2RA).
*Outcomes*: Incidence of GI ulcers or GI symptoms.
*Study design*: Randomized controlled trials (RCTs).

Studies were included irrespective of sample size and the dosage of drugs. Studies not using teprenone as a preventive drug for GI ulcers, studies using combinations of more than one NSAID, and studies using a combination of teprenone with other GI protective agents like PPI/H2RA in the intervention group were excluded. We also excluded non-RCTs, retrospective studies, animal studies, and review articles.

### Data Extraction

A data extraction sheet was prepared for extracting data from the included studies. Two reviewers extracted data independently. Data regarding the first author, publication year, study location, patient population, sample size, demographic details, study and control drug protocol, *Helicobacter pylori* status, study outcomes, and follow-up period were extracted. The outcomes of interest of our review were the incidence of GI ulcers detected on follow-up endoscopy, the incidence of GI symptoms, and the Modified Lanza Score (MLS) if available. Outcome data was fed into meta-analysis software and cross-checked for correctness. We attempted to contact the corresponding author *via* email in case of any missing data. Any other outcomes reported by the included studies were presented in a tabular format.

### Risk of Bias Assessment

The risk of bias was assessed using the Cochrane Collaboration's risk of bias assessment tool-2 by two reviewers independently (15). The following seven domains were used for quality assessment: random sequence generation, allocation concealment, blinding of participants and personnel, blinding of outcome assessment, incomplete outcome data, selective reporting, and other bias. Any disagreements were resolved by discussion. The certainty of the evidence was assessed by the Grading of Recommendations Assessment, Development, and Evaluation (GRADE) tool. The GRADEpro GDT software was used for this purpose [GRADEpro Guideline Development Tool. McMaster University, 2020 (developed by Evidence Prime, Inc.)].

### Statistical Analysis

The software “Review Manager” [RevMan, version 5.3; Nordic Cochrane Center (Cochrane Collaboration), Copenhagen, Denmark; 2014] was used for the meta-analysis. Incidence of GI ulcers and symptoms were summarized using Risk Ratios (RR) with 95% confidence intervals (CI). MLS were pooled using Mean Difference (MD) with 95% CI. Sub-group analyses were carried out for comparing teprenone with control and other drugs. The random-effects model was used for all the meta-analyses. Heterogeneity was assessed using the *I*^2^ statistic. *I*^2^-values of 25–50% represented low, values of 50–75% medium, and more than 75% represented substantial heterogeneity. Due to the inclusion of fewer than 10 studies in the review, funnel plots were not used to assess publication bias.

## Results

**Figure 1:**
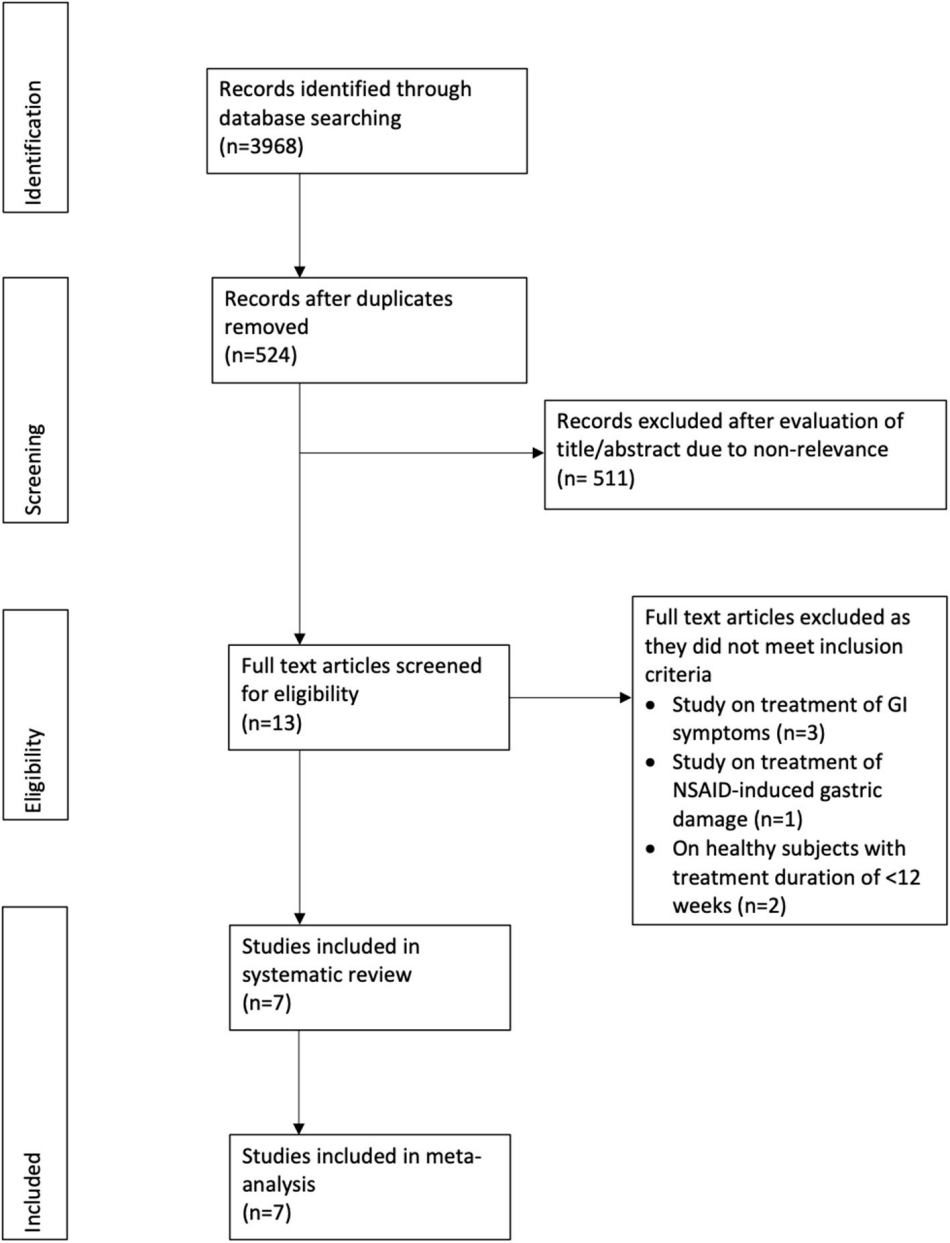
Study flow-chart.

The study flow chart is presented in Figure 1. A total of seven RCTs (16–22) were included in the review (Table 1). Most trials were conducted in China, with one each in Japan (21) and Thailand (18). All trials had excluded patients with GI ulcers and bleeding at baseline. Aspirin was the NSAID used in five trials, one trial (22) used diclofenac while the remaining study (20) used either NSAID or aspirin. The same dosage of teprenone was used in all studies (50 mg thrice daily for a total dose of 150 mg/day). Only one trial (22) compared teprenone with another drug (Famotidine). *Helicobacter pylori* status was not reported in four studies (16, 17, 19, 22). The follow-up ranged from a minimum of 12 weeks/3 months to 12 months.

**Table 1:** Details of included studies.

**References**	**Study location**	**Study population**	**Teprenone dosage**	**Control drug**	**Sample size**	**Mean age (years)**	**Male gender (%)**	**Smokers (%)**	***Helicobacter pylori* status**	**Definition of gastrointestinal ulcer**	**Follow-up**
Chitapanarux et al. (18)	Thailand	Aspirin-naïve patients needing low-dose aspirin for cardiovascular or cerebrovascular disease	50 mg thrice daily	Placebo	Teprenone: 64	59.62 ± 6.35	53.1	12.5	Negative	Defect more than submucosal layer at least 3 mm in diameter in the stomach or duodenum	12 weeks
					Control: 66	58.73 ± 5.48	54.5	18.2	Negative		
Zhao et al. (19)	China	Aspirin-naïve patients needing low-dose aspirin for cardiovascular or cerebrovascular disease	50 mg thrice daily	No drug	Teprenone: 131	65.73 ± 10.30	50.4	19.1	NR	Not defined	3, 6, 12 months
					Control: 136	67.24 ± 9.21	53.7	21.3	NR		
Gong et al. (20)	China	Rheumatic, cardiovascular, cerebrovascular disease patients on long term NSAID or low-dose aspirin	50 mg thrice daily	No drug	Teprenone: 74	54 ± 20	50	16	Negative	Not defined	12 weeks
					Control: 84	55 ± 18	47.6	13.1	Negative		
Takeuchi et al. (21)	Japan	Patients requiring low-dose aspirin for cardiovascular, cerebrovascular, or any disease	50 mg thrice daily	Famotidine 20 mg daily	Teprenone: 28	66.5 ± NR	64.2	25	Positive: 39.2%	Not defined	12 weeks
					Control: 38	72.5 ± NR	57.8	7.8	Positive: 14%		
Xiong et al. (22)	China	Rheumatic disease patients not on NSAIDs for at least 6 months needing diclofenac	50 mg thrice daily	No drug	Teprenone: 21	31 ± 9	42.9	4.8	NR	Mucosal breaks with white or yellow bases sur- rounded by red or pink collars	12 weeks
					Control: 19	31 ± 11	68.4	21.1	NR		
Lu (16)	China	Patients requiring low-dose aspirin for cardiovascular, cerebrovascular, or any disease	50 mg thrice daily	No drug	Teprenone: 42	NR	NR	NR	NR	Not studied	3, 6, 12 months
					Control: 42	NR	NR	NR	NR		
Wu et al. (17)	China	Aspirin-naïve patients needing low-dose aspirin for cardiovascular or cerebrovascular disease	50 mg thrice daily	No drug	Teprenone: 118	69.5 ± 10.4	NR	NR	NR	Not defined	3, 6, 12 months
					Control: 143	69 ± 9.3	NR	NR	NR		

*NSAID, Non-steroidal anti-inflammatory drug; NR, not reported*.

### Meta-Analysis

**Figure 2:**
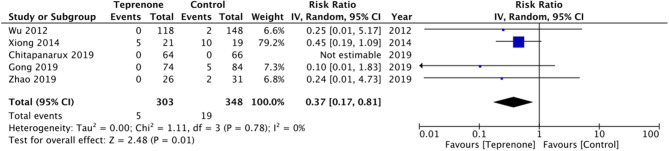
Forest plot for incidence of GI ulcers between teprenone vs. control.

**Figure 3:**
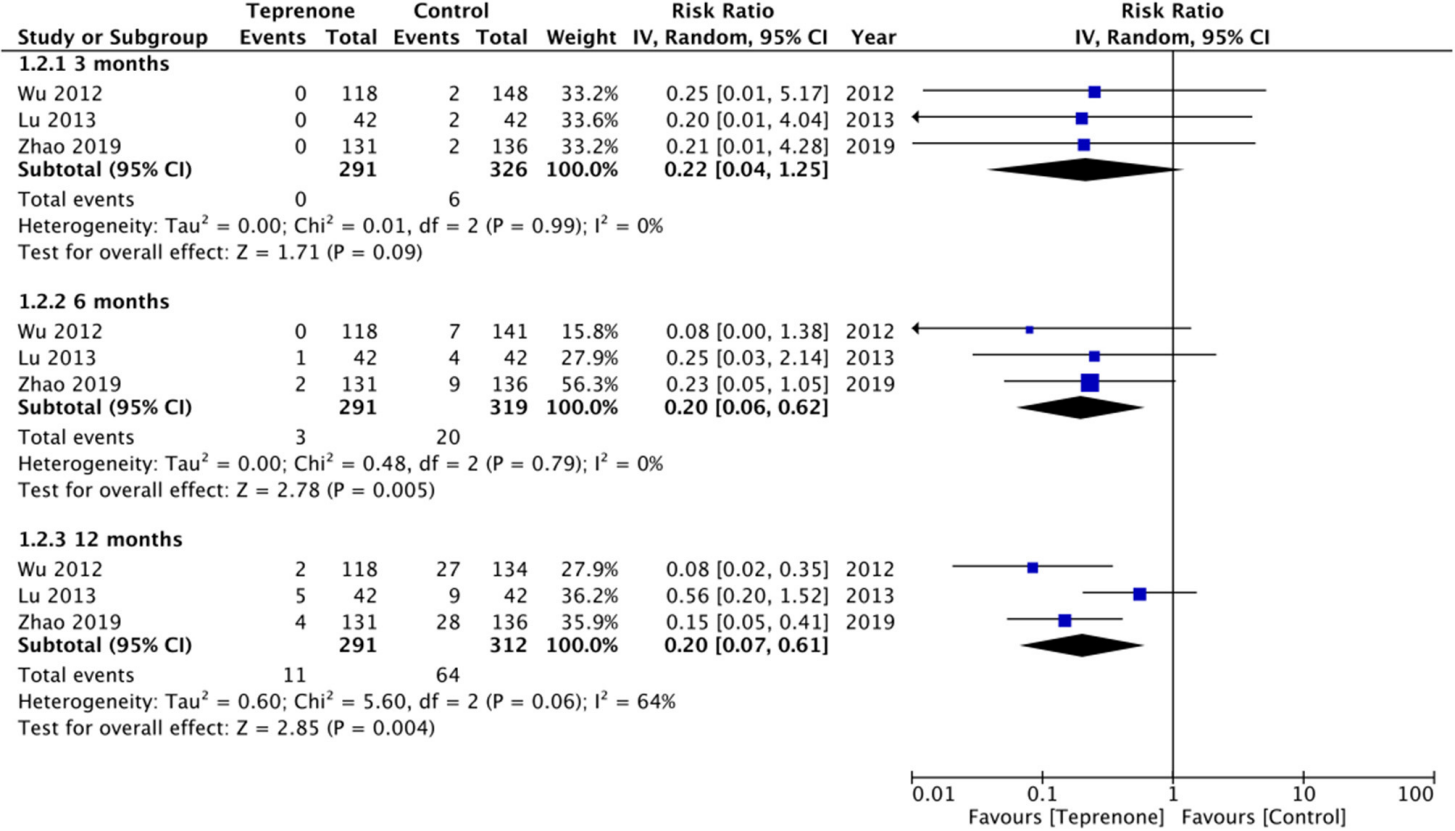
Forest plot for incidence of GI symptoms between teprenone vs. control with sub-group analysis based on duration of follow-up.

**Figure 4:**
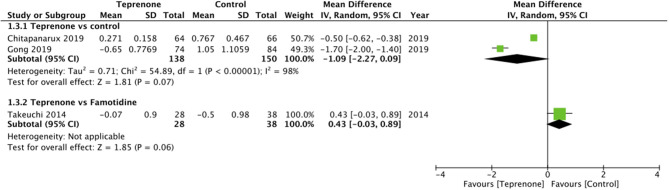
Forest plot for MLS with sub-group analysis based on comparative group.

Of the six trials comparing teprenone with control, four reported incidence of gastric ulcers while one study reported the incidence of small-intestinal ulcers. Meta-analysis indicated a statistically significant reduced risk of GI ulcers in patients receiving teprenone as compared to control after 12 weeks/3 months (RR 0.37 95% CI 0.17, 0.18 *I*^2^ = 0% *p* = 0.01) (Figure 2). The lone trial of Takeuchi et al. (21) comparing teprenone with famotidine reported zero incidences of GI ulcers in both study groups after a follow-up of 12 weeks. The incidence of subjective GI symptoms was pooled from three studies. Our meta-analysis demonstrated no statistically significant difference in the incidence of GI symptoms between the two groups at 3 months (RR 0.22 95% CI 0.01, 1.25 *I*^2^ = 0% *p* = 0.09) but a significant reduction in GI symptoms at 6 months (RR 0.20 95% CI 0.06, 0.62 *I*^2^ = 0% *p* = 0.005) and 12 months (RR 0.20 95% CI 0.07, 0.61 *I*^2^ = 64% *p* = 0.004) in patients on teprenone as compared to control (Figure 3). MLS were reported by just three studies. Pooling data from two studies comparing teprenone with control, our analysis indicated a tendency of non-significant but better reduction in MLS with teprenone (MD −1.09 95% CI −2.27, 0.09 *I*^2^ = 98% *p* = 0.07) (Figure 4). On analysis of data from the study of Takeuchi et al. (21) comparing teprenone with famotidine, the results indicated a tendency of non-significant but better reduction of MLS with famotidine (MD 0.43 95% CI −0.03, 0.89 *p* = 0.06) (Figure 4). The certainty of evidence based on GRADE was deemed to be low for all outcomes (Supplementary Table 2).

### Other Outcomes

Details of all outcomes reported by the included studies along with their results are presented in Table 2. No case of GI bleeding was reported in either the study or control group by four studies (18–21). Xiong et al. (22) used the Lewis score to assess small-intestinal injuries and reported significantly higher scores in the control group.

**Table 2:** Details of outcomes and results reported by the included studies.

**References**	**Outcome**	**Result**
Chitapanarux et al. (18)	Incidence of gastric ulcer	No significant difference between the two groups
	Incidence of GI bleeding	No significant difference between the two groups
	Incidence of gastric erosion	Significantly higher in the placebo group
	Incidence of gastritis	Significantly higher in the placebo group
	Change in MLS	Significantly increased in the placebo group
	Change in GSRS	No difference between the two groups
	Change in COX-1 expression	Significantly higher in the teprenone group
	Adverse events	No significant difference between the two groups
Zhao et al. (19)	Incidence of GI symptoms	Significantly higher in the control group at 6 and 12 months but not at 3 months
	Incidence of gastric ulcer	Significantly higher in the control group at 12 months but not at 3 and 6 months
	Incidence of GI bleeding	No significant difference between the two groups
	Incidence of gastric erosion	No significant difference between the two groups
Gong et al. (20)	Incidence of gastric ulcer	Higher in the control group
	Incidence of GI bleeding	No significant difference between the two groups
	Change in MLS	Significantly increased in the placebo group
	Dyspeptic symptom score	Significantly increased in the placebo group
	Adverse events	No serious adverse events in either groups
Takeuchi et al. (21)	Incidence of gastric ulcer	No significant difference between the two groups
	Incidence of GI bleeding	No significant difference between the two groups
	Change in MLS	Significantly better reduction with famotidine as compared to teprenone
	Incidence of GI symptoms	No significant difference between the two groups
	Adverse events	No significant difference between the two groups
Xiong et al. (22)	Lewis score	Significantly higher in the control group
	Severity of small-intestinal mucosal injuries	Significantly higher in the control group
	Incidence of ulcers and erosions	Significantly higher in the control group
Lu (16)	Incidence of GI symptoms	Significantly higher in the control group
	Fecal occult blood test score	Significantly higher in the control group
	Endoscopy score	Significantly higher in the control group
	Adverse events	No significant difference between the two groups
Wu et al. (17)	Incidence of GI symptoms	Significantly higher in the control group at 3, 6, and 12 months
	Endoscopy score	Significantly higher in the control group at 3, 6, and 12 months

*GSRS, gastrointestinal symptom rating scale; MLS, Modified Lanza score; GI, gastrointestinal*.

No major or serious adverse events were reported in either group by any trial. None of the studies reported any treatment-related discontinuation of any drugs. Only Chitapanarux et al. (18) reported details of specific adverse events. The side-effects reported were nausea (teprenone: 6.25% vs. placebo: 9.09%) and heartburn (teprenone: 9.37% vs. placebo: 9.09%).

### Risk of Bias

**Figure 5:**
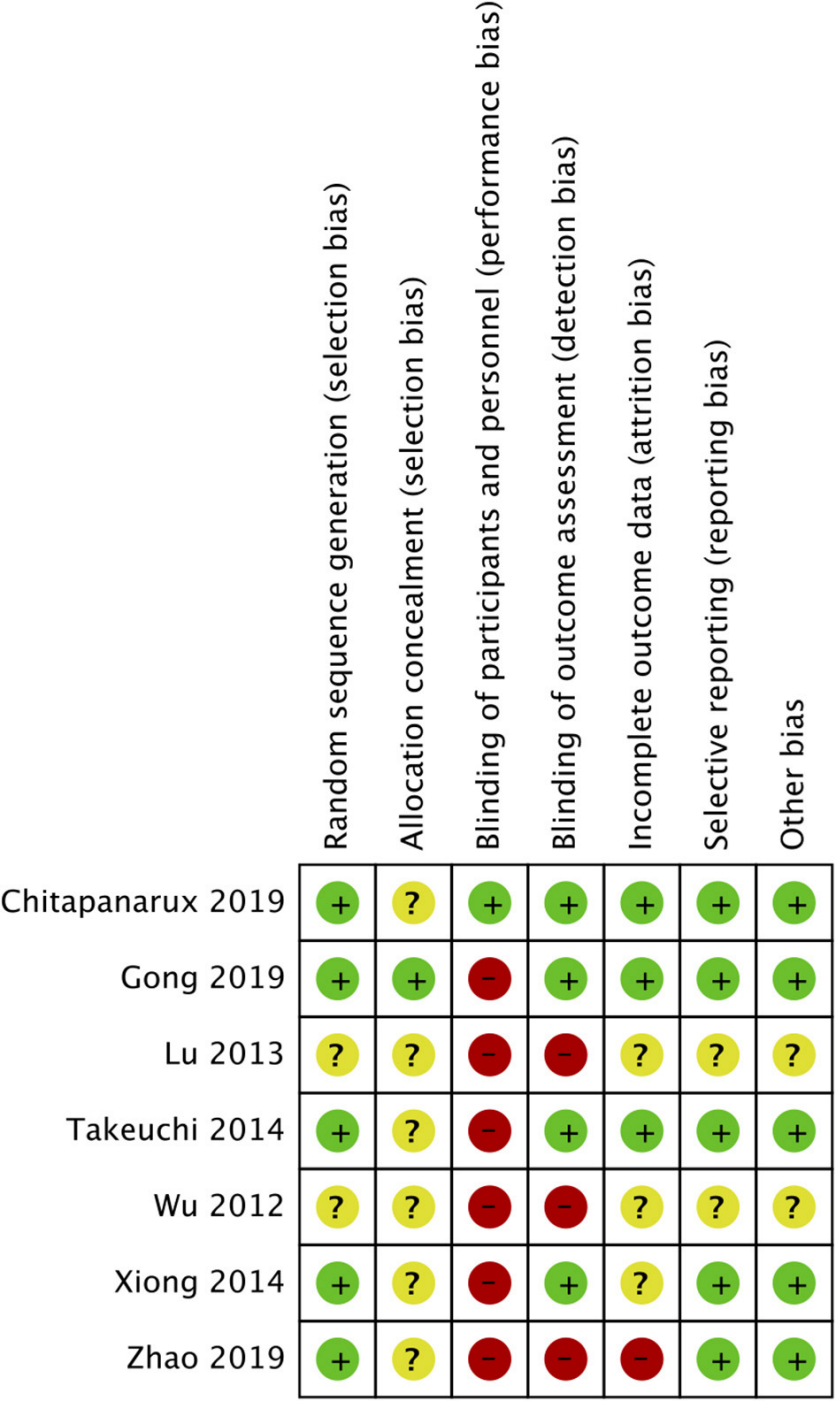
Risk of bias summary.

The risk of bias summary is presented in Figure 5. The majority of the studies did not report adequate information on allocation concealment. Only one trial reported blinding of participants and personnel (18). There was a high risk of bias in blinding of outcome assessment in three studies (16, 17, 19). Attrition bias was high in the study of Zhao et al. (19).

## Discussion

The results of the first systematic review and meta-analysis assessing the preventive efficacy of teprenone against NSAID-induced GI injury presents the following important findings: (1) As compared to placebo or no drug, teprenone significantly reduces the incidence of GI ulcers by 63% at 12 weeks/3 months and reduces the incidence of GI symptoms at 6 months/1 year. (2) On endoscopic evaluation, the degree of GI injury measured by MLS shows a tendency of better reduction by teprenone. (3) The lone RCT comparing teprenone with famotidine, however, demonstrated a tendency of better outcomes with the H2RA. Important to note is the limited number of studies in the meta-analysis and low certainty of evidence on GRADE for all outcomes.

Of available NSAIDs, low-dose aspirin is a valuable anti-thrombotic medication used in several cardiovascular and cerebrovascular diseases. However, the GI adverse effects of even a low dose of the drug are of clinical concern. A number of studies conducted in different geographical regions worldwide have reported the prevalence of endoscopic GI ulcers as well as erosions with low-dose aspirin (23). In one of the largest studies, Uemura et al. (24) detected gastroduodenal ulcers and erosion in 6.5 and 29.2% of Japanese patients on long-term low-dose aspirin. In another multi-centric study, Yeomans et al. (25) reported the prevalence of gastric ulcers to be 10.7% in patients on low-dose aspirin. The prevalence of GI ulcers has varied in different geographical regions with studies reporting prevalence to be as high as 43% in patients receiving long-term NSAIDs, with diclofenac and aspirin being the most common NSAIDs associated with such adverse event (26). Thus, it is important for clinicians using NSAIDs to be aware of such GI injuries and take preventive measures by prescribing gastroprotective medications (27).

In this context, our review is important as it presents level-1 evidence on one such drug i.e., teprenone by pooling evidence from only RCTs. Endoscopic assessment of patients in the study and control groups at 12 weeks/3 months revealed the incidence of GI ulcers to be 1.6 and 5.4%, respectively. On pooling this data for a meta-analysis, our results indicated that teprenone is effective in reducing the incidence of GI ulcers when prescribed with long-term NSAIDs as compared to patients not receiving any drug or placebo. The preventive efficacy of teprenone can be attributed to its unique mechanism of action. It is well-known that inhibition of prostaglandin synthesis is an important component of NSAID-induced gastric injury. Teprenone not only promotes the endogenous synthesis of prostaglandins but also aids in the repair of mucosal integrity by limiting mucosal erosions and increasing mucous secretion without affecting gastric acid secretion (28, 29). This is especially important in the context of small intestinal injuries where the role of gastric acid is limited and decreased mucus secretion and increased mucosal permeability are important factors causing intestinal lesions (30). The trial of Xiong et al. (22) was the lone RCT in our analysis assessing only small intestinal injuries with their results too demonstrating the protective role of teprenone in long-term diclofenac users. However, we were unable to find any more studies assessing the efficacy of teprenone for small intestinal injuries.

An important difference between the seven trials included in our review was the type of NSAID used. Majority studies were conducted on aspirin users while only two studies included patients on other NSAIDs (20, 22). Indeed the use of low-dose aspirin for preventing adverse events in patients with cardiovascular and cerebrovascular disease is much more prevalent than the use of NSAIDs for chronic conditions like rheumatic diseases (1, 2). Literature suggests that the risk of GI injuries varies with the type of NSAID used (31, 32). Owing to the limited number of available studies, it was not possible for this review to separately assess the protective effect of teprenone for different NSAIDs.

Along with NSAID use, infection with *H. pylori* is an important contributor to the etiology of upper GI ulcers (23). Studies have demonstrated that the presence of *H. pylori* can significantly increase the incidence of upper GI mucosal injury (24, 33). Amongst the included studies of our review, only two trials (18, 20) were conducted on *H. pylori* negative individuals with the majority studies not reporting data on *H. pylori* status of included patients. This is an important limitation while assessing results of our review and future studies should take into account this confounding factor to provide robust results. However, since the included studies were RCTs, it may be plausible to assume that the distribution of *H. pylori* positive patients may have been equal in the study and control groups. Furthermore, the two studies (18, 20) pooled for analysis of MLS included only *H. pylori* negative patients in their trials.

A meta-analysis of MLS scores indicated a non-significant difference between teprenone and control. However, considering the 95% CI, there was a tendency for better outcomes with teprenone and the non-significant results may be partly attributed to the limited number of studies available for analysis. While ulcers and bleeding are serious complications with long-term NSAID users, their occurrence is relatively rare, and subjective symptoms of dyspepsia, bloating, etc can be a greater cause of concern from the patient's perspective (23). Our results indicated that subjective symptoms are significantly reduced with long-term use of teprenone. However, only three trials were available for this meta-analysis. It is also important to note that all the three studies were not blinded and subjective symptoms would have been highly influenced due to such bias.

Considering the beneficial effects of teprenone, it would be interesting to compare the efficacy and safety of teprenone with other widely used gastroprotective drugs like PPIs or H2RAs. Our literature search was able to identify only one study comparing teprenone with famotidine with their results demonstrating better outcomes with famotidine. The lower efficacy of teprenone as compared to other drugs has also been demonstrated by studies on patients with dyspeptic symptoms (34, 35). The limited number of studies comparing teprenone with other gastroprotective drugs for NSAID-induced GI damage is a major deficiency in current literature. While any gastroprotective agent can be better than placebo, a good measure of the efficacy of teprenone for NSAID-induced GI injuries can only be provided with its comparison with other established drugs. Only future high-quality RCTs with large sample size comparing teprenone with other PPIs or H2Ras can better elucidate the efficacy of teprenone vis-à-vis other drugs. Such studies should also perform a cost-effective analysis to clarify if teprenone is indeed a cheaper alternative to other drugs.

None of the trials comparing teprenone with placebo or famotidine reported any major adverse events with the use of teprenone. None of the patients discontinued teprenone due to an adverse event. Thus, on analysis of the limited number of RCTs, teprenone seems to be safe. However, further long-term studies with larger sample size shall supplement current evidence.

Our study has some limitations. First, several RCTs had a high or unclear risk of bias in multiple domains. The overall certainty of evidence as assessed by GRADE was found to be low for all outcomes. Secondly, the number of included studies in our review was not high and not all RCTs reported similar outcomes for a meta-analysis. Thirdly, our review included studies reporting both gastric as well as small intestinal lesions. As mentioned earlier, *H. pylori* status was not available from all trials. Furthermore, majority studies did not explicitly define GI ulcers. Difference between studies as to what lesion is classified as an ulcer may have introduced variations in the ulcer incidence between trials. Lastly, all trials were conducted only in a limited number of Asian countries, and this limits the generalization of results to the global population. Furthermore, teprenone is currently available only in limited Asian countries with no record of its availability and cost in European or American markets (36, 37). This is an important factor which limits the research on this drug in only Asian countries.

Nevertheless, ours is the first systematic review and meta-analysis assessing the efficacy of teprenone for preventing GI injuries in long-term NSAID users. A detailed literature search irrespective of language was carried out to include maximum RCTs. A quantitative and qualitative analysis was performed to present compressive evidence comparing teprenone with control as well as other drugs.

To conclude, low-quality evidence indicates a beneficial role of teprenone in preventing GI ulcers and symptoms in patients receiving long-term NSAIDs. Further high-quality RCTs comparing teprenone with placebo as well as other gastroprotective drugs are needed to strengthen current evidence.

## Data Availability Statement

Publicly available datasets were analyzed in this study. This data can be found here: Databases of PubMed, Embase, BioMed Central, CENTRAL, and Google Scholar.

## Author Contributions

CZ conceived, designed the study, and wrote the paper. JW and QX were involved in literature search, data collection, and analyzed the data. QX reviewed and edited the manuscript. All authors read and approved the final manuscript.

## Conflict of Interest

The authors declare that the research was conducted in the absence of any commercial or financial relationships that could be construed as a potential conflict of interest.
