# Supplementary material for: Efficacy of Teprenone for Prevention of NSAID-Induced Gastrointestinal Injury: A Systematic Review and Meta-Analysis
Source: Front Med (Lausanne). 2021 Apr 7;8:647494. doi: 10.3389/fmed.2021.647494 (PMC8058206; doi:10.3389/fmed.2021.647494)
Supplement: Supplementary file 1 [file Table_1.DOCX]

Supplementary Table S1: Search strategy

| **Query** | **Search Details** |
| --- | --- |
| (Geranylgeranylacetone) AND (gastric) | ("geranylgeranylacetone"[Supplementary Concept] OR "geranylgeranylacetone"[All Fields]) AND ("gastrics"[All Fields] OR "stomach"[MeSH Terms] OR "stomach"[All Fields] OR "gastric"[All Fields]) |
| (Geranylgeranylacetone) AND (small intestine) | ("geranylgeranylacetone"[Supplementary Concept] OR "geranylgeranylacetone"[All Fields]) AND ("intestine, small"[MeSH Terms] OR ("intestine"[All Fields] AND "small"[All Fields]) OR "small intestine"[All Fields] OR ("small"[All Fields] AND "intestine"[All Fields])) |
| (Geranylgeranylacetone) AND (injury) | ("geranylgeranylacetone"[Supplementary Concept] OR "geranylgeranylacetone"[All Fields]) AND ("injurie"[All Fields] OR "injuried"[All Fields] OR "injuries"[MeSH Subheading] OR "injuries"[All Fields] OR "wounds and injuries"[MeSH Terms] OR ("wounds"[All Fields] AND "injuries"[All Fields]) OR "wounds and injuries"[All Fields] OR "injurious"[All Fields] OR "injury s"[All Fields] OR "injuryed"[All Fields] OR "injurys"[All Fields] OR "injury"[All Fields]) |
| (Geranylgeranylacetone) AND (ulcer) | ("geranylgeranylacetone"[Supplementary Concept] OR "geranylgeranylacetone"[All Fields]) AND ("ulcer"[MeSH Terms] OR "ulcer"[All Fields] OR "ulcerate"[All Fields] OR "ulcerated"[All Fields] OR "ulcerates"[All Fields] OR "ulcerating"[All Fields] OR "ulceration"[All Fields] OR "ulcerations"[All Fields] OR "ulcerative"[All Fields] OR "ulcers"[All Fields] OR "ulcer s"[All Fields] OR "ulcerous"[All Fields]) |
| (Geranylgeranylacetone) AND (gastrointestinal) | ("geranylgeranylacetone"[Supplementary Concept] OR "geranylgeranylacetone"[All Fields]) AND ("gastrointestinal"[All Fields] OR "gastrointestinally"[All Fields] OR "gastrointestine"[All Fields]) |
| (teprenone) AND (gastric) | ("geranylgeranylacetone"[Supplementary Concept] OR "geranylgeranylacetone"[All Fields] OR "teprenone"[All Fields]) AND ("gastrics"[All Fields] OR "stomach"[MeSH Terms] OR "stomach"[All Fields] OR "gastric"[All Fields]) |
| (teprenone) AND (small intestine) | ("geranylgeranylacetone"[Supplementary Concept] OR "geranylgeranylacetone"[All Fields] OR "teprenone"[All Fields]) AND ("intestine, small"[MeSH Terms] OR ("intestine"[All Fields] AND "small"[All Fields]) OR "small intestine"[All Fields] OR ("small"[All Fields] AND "intestine"[All Fields])) |
| (teprenone) AND (injury) | ("geranylgeranylacetone"[Supplementary Concept] OR "geranylgeranylacetone"[All Fields] OR "teprenone"[All Fields]) AND ("injurie"[All Fields] OR "injuried"[All Fields] OR "injuries"[MeSH Subheading] OR "injuries"[All Fields] OR "wounds and injuries"[MeSH Terms] OR ("wounds"[All Fields] AND "injuries"[All Fields]) OR "wounds and injuries"[All Fields] OR "injurious"[All Fields] OR "injury s"[All Fields] OR "injuryed"[All Fields] OR "injurys"[All Fields] OR "injury"[All Fields]) |
| (teprenone) AND (ulcer) | ("geranylgeranylacetone"[Supplementary Concept] OR "geranylgeranylacetone"[All Fields] OR "teprenone"[All Fields]) AND ("ulcer"[MeSH Terms] OR "ulcer"[All Fields] OR "ulcerate"[All Fields] OR "ulcerated"[All Fields] OR "ulcerates"[All Fields] OR "ulcerating"[All Fields] OR "ulceration"[All Fields] OR "ulcerations"[All Fields] OR "ulcerative"[All Fields] OR "ulcers"[All Fields] OR "ulcer s"[All Fields] OR "ulcerous"[All Fields]) |
| (Teprenone) AND (gastrointestinal) | ("geranylgeranylacetone"[Supplementary Concept] OR "geranylgeranylacetone"[All Fields] OR "teprenone"[All Fields]) AND ("gastrointestinal"[All Fields] OR "gastrointestinally"[All Fields] OR "gastrointestine"[All Fields]) |
