# Supplementary material for: Efficacy of Teprenone for Prevention of NSAID-Induced Gastrointestinal Injury: A Systematic Review and Meta-Analysis
Source: Front Med (Lausanne). 2021 Apr 7;8:647494. doi: 10.3389/fmed.2021.647494 (PMC8058206; doi:10.3389/fmed.2021.647494)
Supplement: Supplementary file 2 [file Table_2.DOCX]

Supplementary Table S2: Assessment of quality of evidence using GRADE

| **Certainty assessment** | | | | | | | **№ of patients** | | **Effect** | | **Certainty** | **Importance** |
| --- | --- | --- | --- | --- | --- | --- | --- | --- | --- | --- | --- | --- |
| **№ of studies** | **Study design** | **Risk of bias** | **Inconsistency** | **Indirectness** | **Imprecision** | **Other considerations** | **New Comparison** | **placebo** | **Relative (95% CI)** | **Absolute (95% CI)** |  |  |
| **GI ulcer** | | | | | | | | | | | | |
| 5 | randomised trials | very serious ^a^ | not serious | not serious | not serious | none | 5/303 (1.7%) | 19/348 (5.5%) | **RR 0.37** (0.17 to 0.81) | **34 fewer per 1,000** (from 45 fewer to 10 fewer) | ⨁⨁◯◯ LOW | CRITICAL |
| **GI symptoms - 3 months** | | | | | | | | | | | | |
| 3 | randomised trials | very serious ^b^ | not serious | not serious | not serious | none | 0/291 (0.0%) | 6/326 (1.8%) | **RR 0.22** (0.04 to 1.25) | **14 fewer per 1,000** (from 18 fewer to 5 more) | ⨁⨁◯◯ LOW | IMPORTANT |
| **GI symptoms - 6 months** | | | | | | | | | | | | |
| 3 | randomised trials | very serious ^b^ | not serious | not serious | not serious | none | 3/291 (1.0%) | 20/319 (6.3%) | **RR 0.20** (0.06 to 0.62) | **50 fewer per 1,000** (from 59 fewer to 24 fewer) | ⨁⨁◯◯ LOW | IMPORTANT |
| **GI symptoms - 12 months** | | | | | | | | | | | | |
| 3 | randomised trials | very serious ^b^ | not serious | not serious | not serious | none | 11/291 (3.8%) | 64/312 (20.5%) | **RR 0.20** (0.07 to 0.61) | **164 fewer per 1,000** (from 191 fewer to 80 fewer) | ⨁⨁◯◯ LOW | IMPORTANT |
| **Lanza - Teprenone vs control** | | | | | | | | | | | | |
| 2 | randomised trials | serious ^c^ | not serious | not serious | serious ^e^ | none | 138 | 150 | - | MD **1.09 lower** (2.27 lower to 0.09 higher) | ⨁⨁◯◯ LOW | CRITICAL |
| **Lanza - Teprenone vs Famotidine** | | | | | | | | | | | | |
| 1 | randomised trials | serious ^d^ | not serious | not serious | serious ^e^ | none | 28 | 38 | - | MD **0.43 higher** (0.03 lower to 0.89 higher) | ⨁⨁◯◯ LOW | IMPORTANT |

**CI:** Confidence interval; **RR:** Risk ratio; **SMD:** Standardised mean difference

#### Explanations

a. Downgraded due to (1) lack of information on allocation concealment in all trials except Gong et al; (2) High risk of bias in blinding of participants and personnel in all trials except Chitapanarux et al; (3) High risk of bias in blinding of outcome assessment in the trials of Wu et al and Zhao et al

b. Lack of blinding in all trials, lack of information on allocation concealment, high attrition in the trial of Zhao et al

c. History of prior use of NSAIDs in the trial of Gong et al. Lack of blinding of participants and personnel in the trial of Gong et al.

d. Lack of information on allocation concealment and lack of blinding of participants and personnel

e. Small sample size and wide confidence interval
